# Compound Odontome: A Case Report

**DOI:** 10.5005/jp-journals-10005-1575

**Published:** 2019

**Authors:** Vivek Rana, Nikhil Srivastava, Noopur Kaushik, Vrinda Sharma, Prerna Panthri, Madan Mohan Niranjan

**Affiliations:** 1–6 Department of Pediatric and Preventive Dentistry, Subharti Dental College and Hospital, Swami Vivekanand Subharti University, Meerut, Uttar Pradesh, India

**Keywords:** Calcified, Compound, Extraction, Mandible, Odontomes

## Abstract

**Introduction:**

Odontomas generally appear as small, solitary, or multiple radio-opaque lesions found on routine radiographic examinations. Traditionally, odontomas have been classified as benign odontogenic tumors and are subdivided into complex or compound odontomas morphologically. Frequently, they interfere with the eruption of the teeth.

**Case report:**

This paper describes the case of a compound odontoma in a 10-year-old boy diagnosed after extraction of the retained right primary mandibular first molar in the radiograph. A surgical excision was performed and the histopathological examination revealed a compound odontome.

**Conclusion:**

Early diagnosis of odontomas and complete removal ensures better prognosis.

**How to cite this article:**

Rana V, Srivastava N, *et al.* Compound Odontome: A Case Report. Int J Clin Pediatr Dent 2019;12(1):64–67.

## INTRODUCTION

The term “odontoma” was first coined by Paul Broca in 1866, who defined the term as tumor formed by the overgrowth of complete dental tissue. Odontomas are developmental anomalies resulting from the growth of completely differentiated epithelial and mesenchymal cells that give rise to functional ameloblast and odontoblast.^1^

Odontomas are nonaggressive hamartomatous developmental malformation or lesions or odontogenic origin which consist of enamel, dentin, cementum, and pulpal tissue.^2^ During odontoma development, enamel and dentin can be deposited in such a way that the resulting structures show anatomically similar to normal teeth structures.^3^

In 1914, odontomes were classified according to their developmental origin as epithelial, composite (epithelial and mesodermal), and connective tissues. According to the WHO classification, odontomes can be divided into three groups such as complex, compound, and ameloblastic fibro-odontomes.^4^

Compound odontomas commonly occur in the incisor-canine region of the maxilla and complex odontomas are frequently located in the premolar and molar region of both jaws.^5^

Radiographically, compound odontomas are characterized by multiple irregular radio-opaque lesions that vary in size and shape and contains tooth-like structures called denticles, whereas complex odontomas manifest as a radiopaque solid mass with occasional nodular elements and surrounded by a fine radiotransparent zone. The lesions are unilocular and are separated from the normal bone by a well-defined corticalization line.^6^

Odontomas generally appear as small, solitary, or multiple radioopaque lesions found on routine radiographic examination. Odontoma may cause disturbances in the eruption of teeth such as impaction, delayed eruption, or retention of primary teeth.^7^

The etiology of an odontoma is not clear,^8^ although local trauma, infection, hereditary anomalies, odontoblastic hyperactivity, or alterations of the genetic components are responsible for controlling tooth development.^9^

Odontomas may be found at any age; however, most of them are detected in first two decades of life. There is no gender predilection and most of the lesions are detected on routine radiographs.^10^ However, Budnick found a slight predilection for the occurrence in males (59%) compared with females (41%). Of all the odontomas combined, 67% occurred in maxilla and 33% in mandible.^11^ The compound odontoma had a predilection for the anterior region, whereas complex odontoma had a predilection for the posterior region of the jaw. Interestingly, both types of odontomas occurred more frequently on the right side of the jaw than on the left side.^12^

## CASE DESCRIPTION

**Fig. 1:**
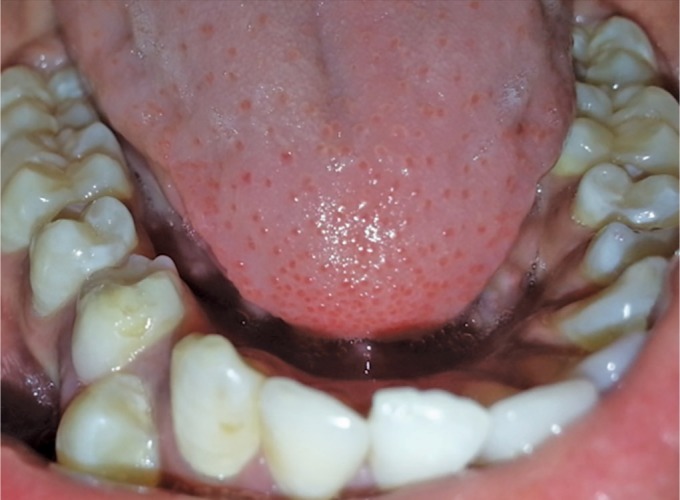
Retained right mandibular primary first molar

A 10-year boy visited the Department of Paedodontics and Preventive Dentistry, Subharti Dental College, with the chief complaint of the retained tooth in the lower right back tooth region. His medical history was non-contributory. There was no history of trauma to his orofacial region. There was no family history of unerupted teeth or hypodontia. On palpation, there was no swelling or tenderness present. He was examined clinically and had all teeth erupted. He was examined clinically and had all permanent teeth erupted with retained right primary mandibular first molar (Fig. 1), while the contralateral tooth, i.e., the mandibular premolars already erupted and were normally positioned in the arch, on the contrary, the right mandibular premolar erupted buccally. There was no history of extraction of the tooth.

**Fig. 2:**
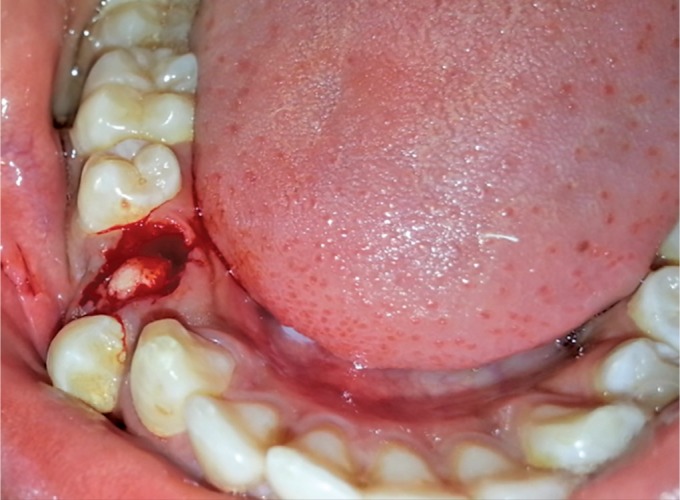
Calcified structures into the extracted socket

The extraction of the retained tooth was planned. After extracting the same, small calcified structures were observed (Fig. 2) in the extracted socket.

**Fig. 3:**
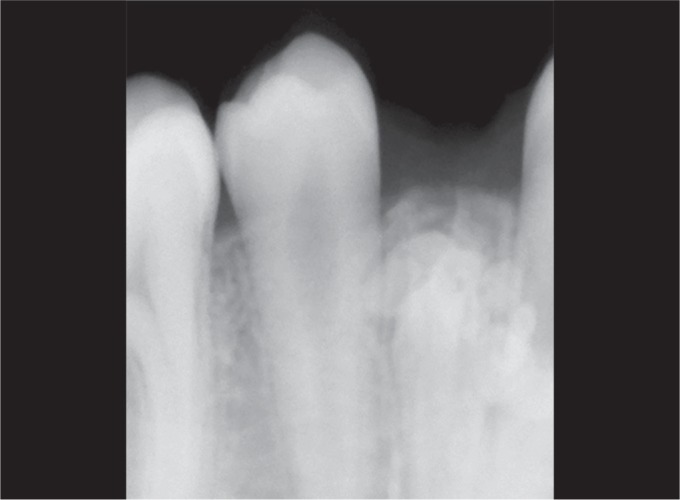
Calcified radiopaque structures

Intraoral periapical radiograph (IOPA) revealed approximately 8–10 calcified or radiopaque structures which were similar to teeth and were located between canine and second premolar (Fig. 3).

**Fig. 4:**
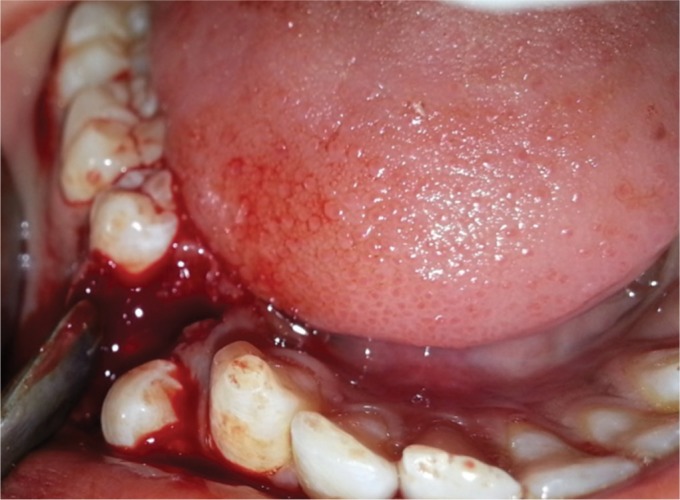
Elevation of the flap

**Fig. 5:**
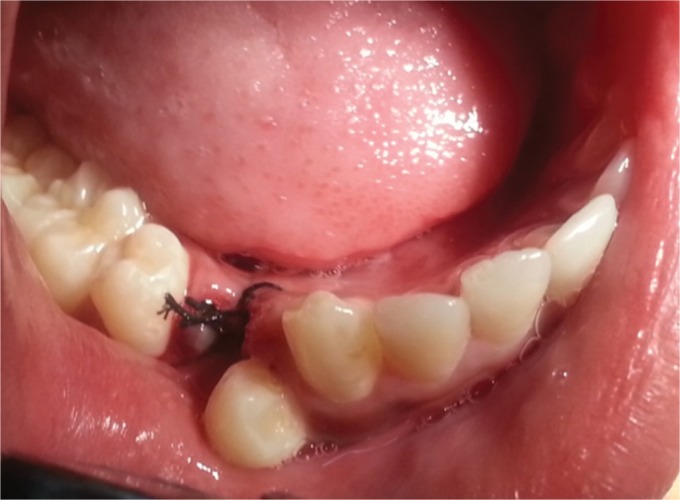
Wound closure by 3-0 silk suture

These structures present with a well-defined radiopacity situated in the bone. These are extending from just below the crest toward at the level of apex of the adjacent teeth. After the extraction of the primary teeth, two vertical incisions were given followed by the elevation of the flap (Fig. 4), and all the calcified tooth-like structures were surgically removed. Finally, the wound was closed by the 3-0 silk suture (Fig. 5).

**Fig. 6:**
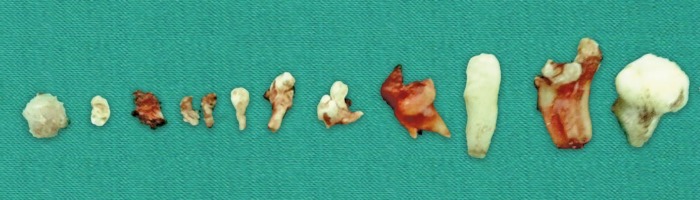
Calcified tooth-like structures

Gross as well as radiographic examination showed many miniature teeth-like structures (Fig. 6), and these structures were sent for histopathological examinations.

**Fig. 7:**
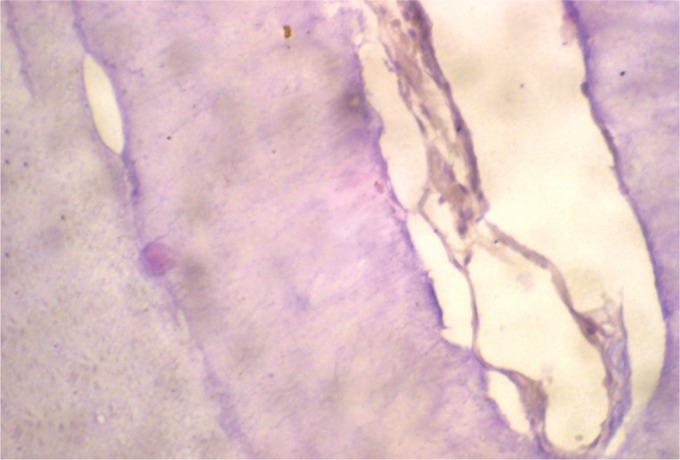
Histopathological section

Decalcified sections of miniature tooth-like structures stained with hematoxylin and eosin showed the regularly arranged dental hard tissue resembling the tubular dentin with minimal fibrocellular connective tissue stroma (Fig. 7).

**Fig. 8:**
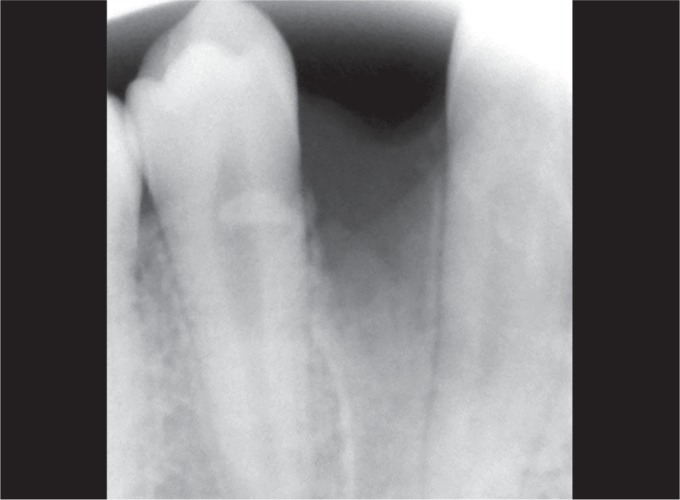
Postoperative intraoral periapical radiograph

Based on clinical, radiographic, and histopathologic features, it was suggestive of compound odontome. The postoperative radiograph showed complete excision of odontomas and wound healing was satisfactory without any complications (Fig. 8).

## DISCUSSION

Odontomas are relatively common, asymptomatic odontogenic hamartomatous malformations. The most common clinical presentation for an odontoma is the association with impacted or retained primary teeth.^12^

The mean age of detection on an average is 14.8 years, with the prevalent age being the second decade of life.

There is a slight predilection for the occurrence in males (59%) as compared with females (41%). The compound odontome is known to occur more commonly in the maxilla (67%) than the mandible (33%), with a marked predilection for the anterior maxillary region (61%).^8,13,14^

In 1914, Gabell, James, and Payne grouped odontome according to their developmental origin: epithelial, composite (epithelial and mesodermal), and mesodermal.^1^

In 1946, Thoma and Goldman gave a classification which is as follows:^15^

- Geminated composite odontomes: two or more, more or less well-developed teeth fused together.
- Compound composite odontomes: made up of more or less rudimentary teeth.
- Complex composite odontomes: calcified structure bearing no great resemblance to the normal anatomical arrangement of dental tissues.
- Dilated odontomes: the crown or root part of tooth shows a marked enlargement.
- Cystic odontomes: an odontome that is normally encapsulated by fibrous connective tissue in a cyst or in the wall of a cyst.

According to the World Health Organization (WHO) classification, odontomes can be divided into three groups:^16^

1. Complex odontome: when the calcified dental tissues are simply arranged in an irregular mass bearing no morphologic similarity to rudimentary teeth.
2. Compound odontome: composed of all odontogenic tissues in an orderly pattern, which result in many teeth-like structures, but without morphological resemblance to normal teeth.
3. Ameloblastic fibro-odontome: consists of varying amounts of calcified dental tissue and dental papilla-like tissue, the later component resembling an ameloblastic fibroma. The ameloblastic fibro-odontome is considered as an immature precursor of complex odontoma.

Compound odontomas show a high degree of morpho-differentiation, resulting in a lesion consisting of many tooth-like structures generally enclosed in a fibrous capsule.^9^

The radiographic findings of odontomas depend on their stage of development and degree of mineralization. The first stage is characterized by radiolucency due to the lack of calcification. Partial calcification is observed in the intermediate stage, while in the third stage, the lesion usually appears as radiopaque masses surrounded by radiolucent areas corresponding to the connective tissue histologically.^17^ In our case, compound odontome was found in the posterior region which is rare with an incidence of 54% in the region of the maxilla and 26.2% in the posterior region.^18^ The absolute incidence of odontogenic tumors of the jaws varies from 0.02 to 0.1%, out of which, odontomas constitute about 22%.^19^

The diagnosis is usually established on the basis of routine radiological examination (panoramic and/or intraoral radiographs), or on evaluating the cause of delayed tooth eruption.^20^ The treatment of choice is surgical removal of the lesion in all cases, followed by the histopathological study to confirm the diagnosis.

Odontomas are treated by conservative surgical removal and there is little probability of recurrence.^21^ Timely detection and surgical enucleation of odontoma followed by curettage is recommended to prevent complications such as tooth loss, cystic changes, bone expansion, and delayed eruption of permanent teeth.^22^

## CONCLUSION

Odontomas rarely erupt into the mouth and tend to be associated with impacted as well as retained teeth. A thorough visual, manual as well as radiographic examination should be performed on all pediatric patients who present with clinical evidence of delayed eruption or missing tooth. Also, early diagnosis of odontomas allows the adoption of a less-complex and less-expensive treatment and ensures better prognosis.
